# Bridging the global divide: Strengthening translational autophagy research in cardiovascular disease for low and middle-income countries

**DOI:** 10.21542/gcsp.2025.20

**Published:** 2025-05-15

**Authors:** Ornella Fiorillo-Moreno, Jessica Manosalva-Sandoval, Maria Isabel Barraza Viloria

**Affiliations:** 1 Clínica Iberoamérica, Barranquilla, Colombia; 2 Universidad de la Costa, CUC, Barranquilla, Colombia

Cardiovascular diseases are the leading cause of morbidity and mortality globally^1^. However, this impact is more pronounced in low- and middle-income countries (LMICs), where population aging has been more rapid than that in high-income countries^1^, and unhealthy lifestyles have contributed to a higher incidence of pathological health phenotypes in older adults. As a result, the rising trend in morbidity and mortality from cardiovascular, metabolic, and cerebrovascular diseases is expected to continue^1^. In this context, it has been proposed that understanding biological variables, supported by the use of omics technologies, could drive precision medicine and provide valuable insights for translation into public policies, scientific advancements, and evidence-based decision-making. However, achieving this requires significant progress in translational research on cardiovascular diseases within LMICs^2^.

A global bibliometric analysis was recently published^3^, in which the authors thoroughly and insightfully explored the evolution of research on autophagy in cardiomyopathies, reflecting the progress in this area of translational research in cardiovascular diseases, one of the most prevalent conditions worldwide^3^. While the authors highlight the scientific impact, collaborations, and potential gaps that could strengthen future research directions, it is remarkable that there are additional highly relevant implications that could further enhance the discussion and interpretation of this study’s findings. Notably, while the study includes data from 70 countries, its Table 1 shows that none of the top 10 contributing countries are classified as LMICs by the World Bank, and the cooperative network illustrated in Figure 3C lacks representation from Africa and most of Latin America. This further substantiates the concern that the study’s conclusions predominantly reflect the output and collaboration of high-income economies.

Although the authors mentioned the countries and institutions that have led research on autophagy in cardiomyopathies (as a significant example of translational research in cardiovascular diseases), they predominantly highlighted China and the United States—developed countries with robust science and technology infrastructure and substantial research resources^3^. However, compared with these leading countries, there is no specific focus on regions or countries where research is scarce, which represents a crucial niche for discussing the availability of translational evidence, scientific policies, and future opportunities for funding and research prioritization.

The translational science model has historically proven to be the most valuable for testing relevant hypotheses in biomedical sciences, with clinical applications, and for improving decision-making precision in healthcare^4,5^. This observation holds when considering that countries with more intensive translational research efforts have access to a greater number of innovative technological tools, facilitating the discovery of new therapeutic targets, drug design, biomedical device creation, and the development of precision medicine strategies for estimating population risks^4,5^; for example, endemic conditions such as Chagas disease in Latin America, nutritional cardiomyopathies in Sub-Saharan Africa, and the increasing burden of diabetic cardiomyopathy across South Asia represent context-specific translational priorities^1,2^.

The use of in vivo models to prospectively investigate tissue, structural, and organ reactivity with human-like similarity is one of the most well-known and widely used experimental tools in cardiovascular disease research^6,7^. However, these models are less frequently replicated in laboratories in LMICs because of high infrastructure and maintenance costs, technical complexity, and regulatory hurdles, which have been widely recognized as barriers to implementing high-fidelity animal models in LMICs cardiovascular research settings^4,5^.

The international collaborations described, as well as the most prolific authors on autophagy in cardiomyopathies^3^, serve as benchmarks and potential solutions for institutions in LMICs to engage in international consortia or establish new high-value scientific collaborations in translational research on cardiovascular diseases. Such partnerships can be facilitated through mechanisms such as the NIH Fogarty International Center, Wellcome Trust’s LMICs-focused programs, or regional networks such as the African Research Universities Alliance (ARUA) and Latin American Network of Cardiovascular Research. These collaborations should promote interdisciplinarity and transdisciplinary research, ultimately yielding valuable new findings.

It is also important to recognize the considerable heterogeneity within LMICs, which includes countries with rapidly growing research infrastructures as well as those still facing fundamental structural challenges. With the growing emphasis on prioritizing quality over the quantity of medical research^8^ and the increasing utilization of molecular and computational research with potential clinical applicability in developing countries^9^, future bibliometric studies should equally highlight the significant gaps and absence of research lines identified as priorities in global or public health in specific regions. All of this should be grounded in rigorous scientific standards and research relevance aimed at ultimately improving the quality of evidence and availability of real-world primary data^10^. These types of studies provide a comprehensive overview of scientific advancements, gaps, and opportunities, which are invaluable for scientific discourse, particularly in light of translational research on cardiovascular diseases in regions where insufficient studies have been conducted to address widespread public health priorities.

## Ethical Approval

Not applicable.

## Competing interests

The author(s) declare no conflict of interest in preparing this article.

## Authors’ contributions

All authors contributed equally to this manuscript.

## Funding

No funding was received for this study.

## Availability of data and materials

Not applicable.
